# Efficacy of epidermal growth factor receptor-tyrosine kinase inhibitor for lung adenosquamous cell carcinoma harboring EGFR mutation: a retrospective study and pooled analysis

**DOI:** 10.3389/fonc.2024.1354854

**Published:** 2024-07-04

**Authors:** Xueming Xia, Wei Du, Yan Zhang, Yanying Li, Min Yu, Yongmei Liu

**Affiliations:** ^1^ Division of Head & Neck Tumor Multimodality Treatment, Cancer Center, West China Hospital, Sichuan University, Chengdu, China; ^2^ Department of Targeting Therapy & Immunology, Cancer Center, West China Hospital, Sichuan University, Chengdu, China; ^3^ Division of Thoracic Tumor Multimodality Treatment, Cancer Center, West China Hospital, Sichuan University, Chengdu, China

**Keywords:** tyrosine kinase inhibitor, adenosquamous lung carcinoma, EGFR, mutation, lung cancer

## Abstract

**Objectives:**

To explore the efficacy of epidermal growth factor receptor tyrosine kinase inhibitors (EGFR-TKIs) on lung adenosquamous cell carcinoma (ASC) with EGFR mutation.

**Methods:**

Efficacy of EGFR-TKIs in the treatment of advanced or recurrent lung ASC with EGFR mutations was assessed retrospectively in 44 patients. Pooled analysis of 74 patients using EGFR-TKIs, including 30 patients selected from 11 publications, was conducted.

**Results:**

In our retrospective research, patients treated with EGFR-TKI in ASC with EGFR mutations had objective response rate (ORR) of 54.5%, disease control rate (DCR) of 79.5%, median progression free survival (mPFS) of 8.8 months, and median overall survival (mOS) of 19.43 months, respectively. A pooled analysis reveals ORR, DCR, mPFS, and mOS are, respectively, 63.4%, 85.9%, 10.00 months, and 21.37 months for ASC patients. In patients with deletions in exon 19 and exon 21 L858R mutations, mPFS (11.0 versus 10.0 months, P=0.771) and mOS (23.67 versus 20.33 months, P=0.973) were similar. Erlotinib or gefitinib-treated patients had an overall survival trend that was superior to that of icotinib-treated patients.

**Conclusions:**

ASC harboring EGFR mutations can be treated with EGFR-TKI in a similar manner to Adenocarcinoma (ADC) harboring EGFR mutations. There is still a need for further investigation to identify the separate roles of ASC’s two components in treating EGFR.

## Introduction

Lung adenosquamous cell carcinoma (ASC)has a low incidence of about 0.4%-4%, making it one of the rarest types of lung cancer (1). ASC is characterized by the presence of both glandular and squamous components, each constituting at least 10% of the tumor (2). This dual histology contributes to the aggressive nature of ASC and poses significant therapeutic challenges. The prognosis for ASC patients is generally poorer compared to those with pure adenocarcinoma or squamous cell carcinoma, reflecting its more aggressive biological behavior and limited treatment options​. Although immunotherapy improves survival of ASC patient, compared to squamous cell carcinoma and adenocarcinoma, ASC patients have a worse prognosis (3–5).

In patients with non-small cell lung cancer (NSCLC) harboring epidermal growth factor receptor (EGFR) mutations, treatment with EGFR tyrosine kinase inhibitors (EGFR-TKIs) are now norm. Third-generation EGFR-TKIs, such as osimertinib, have become the current standard of care, particularly for patients with EGFR T790M resistance mutations (6, 7). EGFR mutations are predominantly found in adenocarcinoma, but they can also be detected in 54.8% of ASC patients (8). Despite the proven efficacy of EGFR-TKIs in treating EGFR-mutant NSCLC, the evidence for their effectiveness in ASC is limited due to the rarity of the condition and the consequent scarcity of comprehensive studies (9). Current treatment guidelines and clinical trials predominantly focus on adenocarcinoma, leaving a gap in tailored therapeutic strategies for ASC patients with EGFR mutations.

In this context, our study aims to explore the efficacy of EGFR-TKIs in patients with EGFR-mutant ASC through a retrospective analysis and pooled data from published studies. We seek to provide insights into the clinical outcomes and potential benefits of EGFR-TKIs therapy in this unique patient population, thereby addressing a critical gap in the management of ASC.

## Patients and methods

### Patients

Between January 2009 and April 2022, we collected clinical data on ASC patients treated with EGFR-TKI at West China Hospital. All patients underwent bronchofiberscope or percutaneous lung biopsies, followed by immunohistochemistry (IHC) for pathological confirmation. These retrospective analyses were carried out with informed consent from each patient.

We searched PUBMED for all publications describing the use of EGFR-TKI in advanced or recurrent EGFR mutant ASC patients for further research into its efficacy. There were three subject headings used during the search period of 2005 to 2022: lung cancer, mutation, and EGFR. Journals and publications were not limited by the strategy, but abstracts of conferences were not accepted. An evaluation of EGFR-TKIs used to treat advanced or recurrent ASC patients harboring EGFR mutations was included in this study. The choice was limited to researches published in the English journal. EGFR-TKI therapy was offered to patients who met all three criteria: (1) advanced or recurrent ASC, (2) EGFR mutation, and (3) acceptance of EGFR-TKI therapy (erlotinib 150mg/day, gefitinib 250mg/day, icotinib 125mg tid or dacomitinib 45mg/day). Data, such as EGFR mutation type, EGFR-TKI line, and treatment with EGFR-TKI, were collected as baseline factors. The authors were asked for data that wasn’t included in the article.

### Test method for EGFR mutation

In the retrospective data, tissues that were embalmed or freshly harvested were used to extract DNA. The mutations in EGFR were identified using a quantitative PCR analysis using the Amplification Refractory Mutation System.A EGFR mutation was detected using the protocol represented in each study, according to the published data.

### Clinical assessments

The Response Evaluation Criteria in Solid Tumors were used to evaluate the efficacy of the EGFR-TKI targeted therapy. There were four types of responses: progressive disease (PD), stable disease (SD), partial response (PR), and complete response (CR). The objective response rate (ORR) is determined by dividing the percentage of patients who were CR or PR by all patients. CR, PR, and SD patients were divided by total patients to determine the disease control rate (DCR). A prognosis of progression free survival (PFS) was calculated from the beginning of treatment to the onset of PD. We also calculated overall survival (OS) from the moment treatment began until death. It was on July 22, 2022, that the last follow-up visit was carried out. In statistical analysis, patients who did not progress or were alive were censored on July 22, 2022.

### Statistical methods

Qualitative variables were illustrated as the way of absolute and percentage amounts, while continuous variables were illustrated as medians with ranges. In order to conduct the survival analysis, Kaplan-Meier methods were used. An univariate analysis of log-rank tests was performed in order to determine which prognostic factors affect PFS and OS. A multivariate analysis was conducted by using Cox regression. The significance of P values is determined by using 0.05. Analyses were conducted using SPSS version 22.0.

## Results

### Patient characteristics

EGFR-TKI treatment was administered to 44 ASC patients with EGFR mutations at the two cancer centers for the purposes of assessing efficacy. Of the 44 ASC patients, there were 22 females and 22 males. Age range was 34-82 years (median 60.5 years). There were 17 patients with a history of smoking. Among the patients, 20 had a mutation in exon 19 (19-DEL), 21 had a mutation in exon 21 (L858R), while 3 had a rare sensitive mutation (G719X, L861Q). As a first-lines treatment, 27 patients were treated with EGFR-TKI, and 17 patients were treated in a second or more line of treatment. There were 11 patients treated with erlotinib, 21 patients treated with gefitinib, 11 patients treated with icotinib, and 1 patient treated with dacomitinib (**Table 1**).

**Table 1:** Clinical characteristics of ASC patients with EGFR mutation.

Characteristics	Study data (n=44)	Published data(n=30)	Total data (n=74)
Age (years)	60.5(34-82)	57(30-76)	58.5(30-82)
Age	44	21	65
<60	22(33.8%)	12(18.5%)	34(52.3%)
≥60	22(33.8%)	9(13.9%)	31(47.7%)
Gender	44	30	74
Male	22(29.7%)	11(14.9%)	33(44.6%)
Female	22(29.7%)	19(25.7%)	41(55.4%)
Smoking status	44	28	72
Smoker (current/former)	17(23.6%)	5(7.0%)	22(30.6%)
Non-smoker (never)	27(37.5%)	23(31.9%)	50(69.4%)
EGFR mutation type	44	30	74
19 Del	20(27.0%)	20(27.0%)	40(54.0%)
21 L858R	21(28.4%)	8(10.8%)	29(39.2%)
G719X, L861Q	3(4.0%)	1(1.4%)	4(5.4%)
21L858R and T790M	0(0.%)	1(1.4%)	1(1.4%)
Lines of EGFR-TKI	44	22	66
1ST line	27(40.9%)	10(15.2%)	37(56.1%)
2nd+ line	17(25.8%)	12(18.1%)	29(43.9%)
EGFR-TKI treatment	44	23	67
Erlotinib	11(16.4%)	14(20.9%)	25(37.3%)
Gefitinib	21(31.4%)	9(13.4%)	30(44.8%)
Icotinib	11(16.4)	0(0%)	11(16.4%)
Dacomitinib	1(1.5%)	0(0%)	1(1.5%)

ASC, adenosquamous cell carcinoma; EGFR, epidermal growth factor receptor; TKI, tyrosine kinase inhibitor.

### Efficacy of EGFR-TKI

**Figure 1:**
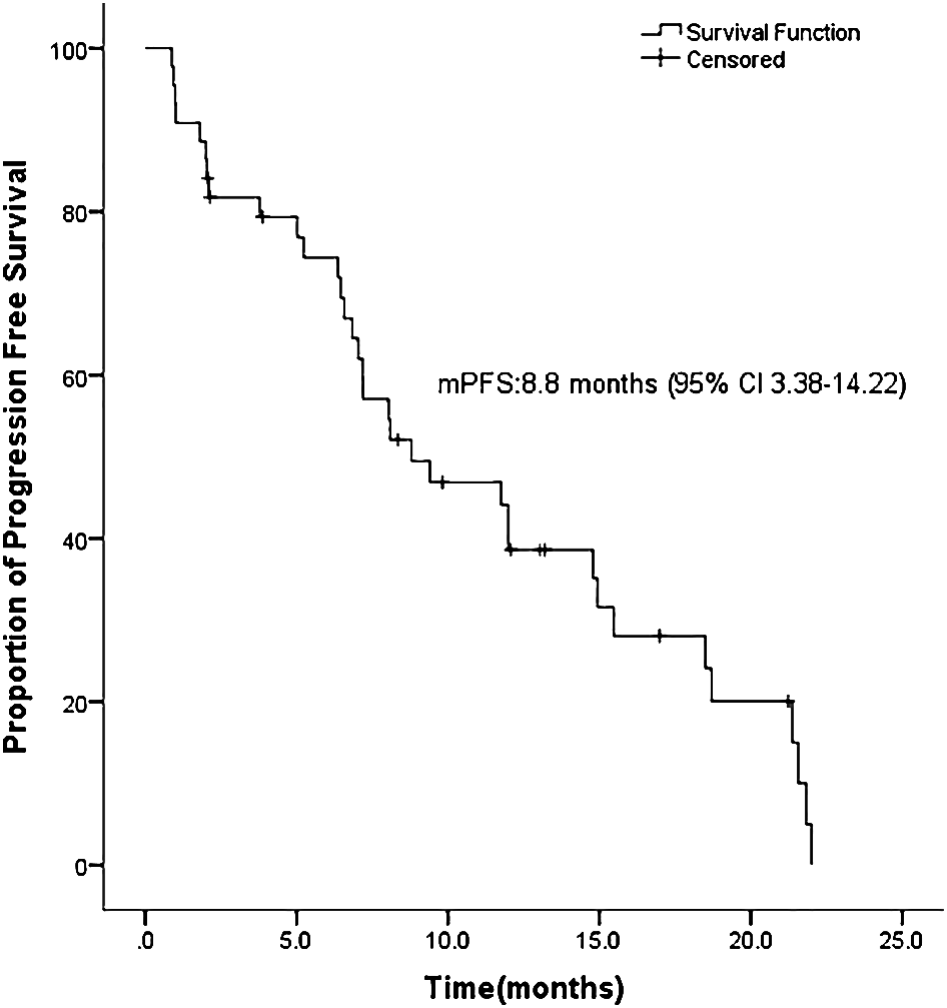
Progression free survival (PFS) of ASC in our bicenter research.

**Figure 2:**
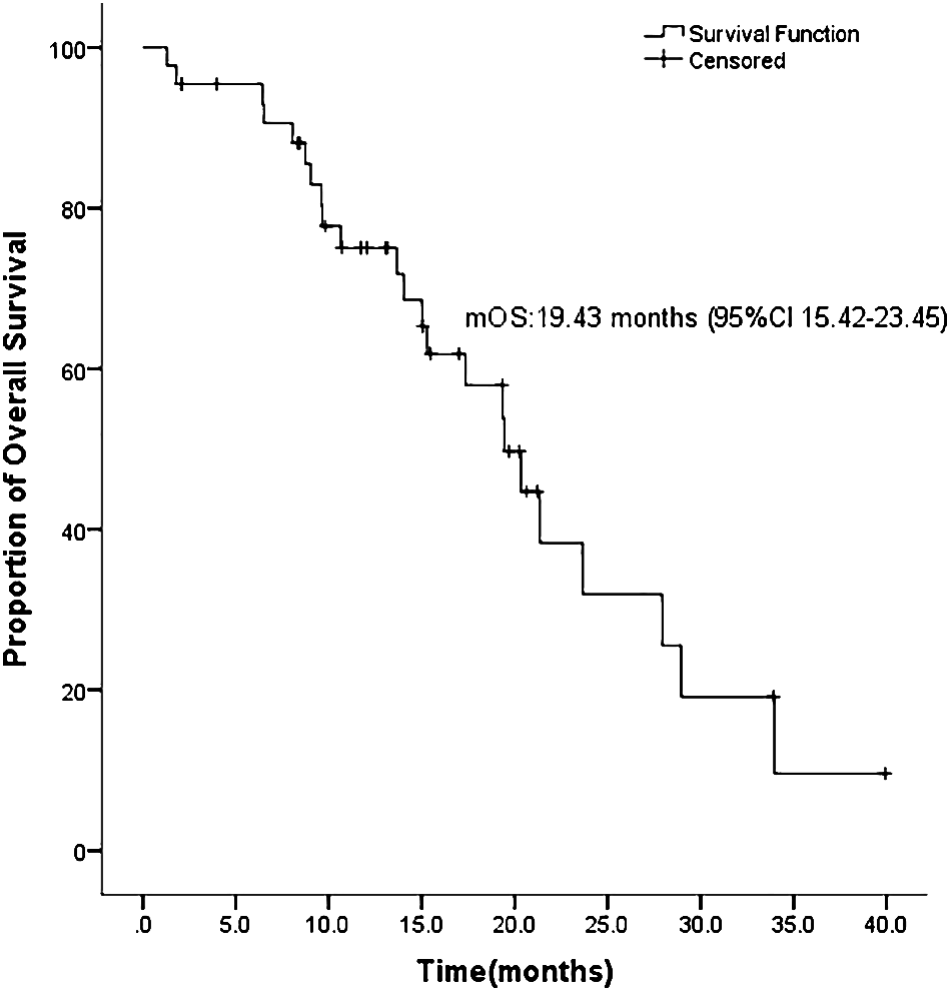
Overall survival (OS) of ASC in our bicenter research.

ASCs with EGFR mutations responded to EGFR-TKI with 2 CRs, 22 PRs, 11 SDs, and 9 PDs. The ORR was 54.5% and the DCR was 79.5% for the 44 patients (**Table 2**). Ten patients had not yet progressed, while 21 patients were still alive on July 22, 2022. **Figure 1** shows the mPFS was 8.8 months (95% CI 3.38-14.22), and **Figure 2** shows the mOS of 19.43 months (95% CI 15.42-23.45).

**Table 2:** Best response to EGFR-TKI in ASC patients.

Best response	Study data (n=44)	Published data(n=30)	Total data(n=74)
Complete response (CR)	2	1	3
Partial response (PR)	22	20	42
Stable disease (SD)	11	5	16
Progressive disease (PD)	9	1	10
Objective response rate (ORR)	54.5% (24/44)	77.8% (21/27)	63.4% (45/71)
Disease control rate (DCR)	79.5% (35/44)	96.3% (26/27)	85.9% (61/71)

ASC, adenosquamous cell carcinoma; EGFR, epidermal growth factor receptor; TKI, tyrosine kinase inhibitor.

### Pooled analysis

A total of 30 patients who met the inclusion criteria from eleven research studies were included in this study (10–20). The 11 researches consisted of 8 retrospective studies and 3 case reports. A total of eight of these researches were conducted in East Asian countries. Thirteen patients were from western countries. Definitive data of age, gender, smoking status, EGFR mutation type, lines of EGFR-TKI and EGFR-TKI treatment could be extracted in 21 (70.0%), 30 (100.0%), 28 (93.3%), 30 (100.0%), 22 (73.3%) and 23 (76.7%) of the 30 patients, respectively (**Tables 1**, **3**, **4**).

**Table 3:** The 11 published reports which we could extract the data of recurrent or advanced ASC patients who had EGFR mutation and were treated with EGFR-TKI.

Author	Year published	Study design	Country of origin	NO. ASC patients Harboring EGFR mutation
Tokumo et al. (10)	2005	Retrospective trial	Japan	1
Ichihara et al. (11)	2007	Retrospective trial	Japan	1
Xu et al. (12)	2009	Retrospective trial	China	1
Paik et al. (13)	2012	Retrospective trial	America	9
Iwanaga et al. (14)	2012	CASE REPORT	Japan	1
Cho et al. (15)	2012	Retrospective trial	Korea	3
Baik et al. (16)	2013	CASE REPORT	America	2
Inoue et al. (17)	2013	Prospective trial	Japan	2
Powrozek et al. (18)	2014	Prospective trial	Poland	2
Tamura et al. (19)	2015	CASE REPORT	Japan	1
Song et al. (20)	2013	Prospective trial	China	7

ASC, adenosquamous cell carcinoma; EGFR, epidermal growth factor receptor; TKI, tyrosine kinase inhibitor.

**Table 4:** Individual patient data of the ASC patients with EGFR mutations extracted from the 11 studies that evaluated the efficacy of EGFR-TKI for ASC patients with EGFR mutations.

Author	No.	mutation	Age(y)	Sex	PS	Smoking	Line	TKI	Response	PFS (m)	OS (m)
Tokumo et al. (10)	1	L858R	77	F	1	–	2	G	SD	–	–
Ichihara et al. (11)	2	L858R+T790M	–	F	–	–	–	G	SD	1.6	8.7
Xu et al. (12)	3	L858R	–	M	–	Y	≧2	G	PR	5.3+	5.3+
Paik et al. (13)	4	19-del	61	M	–	N	2	E	PR	12.1	27.5
	5	19-del	71	F	–	N	1	E	–	19.6	32.9+
	6	19-del	58	F	–	N	2	E	SD	23.6	32.2+
	7	19-del	45	F	–	N	2	E	–	–	15.9
	8	19-del	46	M	–	N	1	E	PR	5+	6.6+
	9	19-del	73	M	–	Y	3	E	–	–	29.8
	10	19-del	76	M	–	N	1	E	PR	5.3	5.3
	11	L858R	30	F	–	N	2	E	PR	8.4	10.9+
	12	19-del	50	M	–	N	1	E	PR	9.2+	9.6+
Iwanaga et al. (14)	13	19-del	56	F	–	Y	2	G	CR	36.0	–
Cho et al. (15)	14	19-del	48	F	–	N	2	G	PR	4.53	16.93
	15	19-del	43	F	–	N	2	E	PR	8.23	24.03
	16	19-del	51	F	–	N	2	E	PR	13.53	25.0
Baik et al. (16)	17	L858R	53	F	–	N	1	E	PR	9.0	19.0
	18	19-del	61	F	–	N	1	E	PR	4+	–
Inoue et al. (17)	19	19-del	67	F	0	N	1	G	SD	2.97+	36.0
	20	L858R	60	F	0	Y	1	G	PR	10.0	32.8+
Powrezek et al. (18)	21	19-del	58	M	2	N	1	G	PR	11.0	16.0
	22	L861Q+G719X	51	M	1	N	1	E	PR	6+	8+
Tamura et al. (19)	23	19-del	66	F	1	N	2	G	PR	9.0	–
Song et al. (20)	24-30	19del(4)/L858R(3)	–	F(4)/M(3)	–	Y(1)/N(6)	–	E/G	PR(5)/SD(1)/PD(1)	m8.7	–

ASC, adenosquamous cell carcinoma; EGFR, epidermal growth factor receptor; TKI, tyrosine kinase inhibitor; CR, complete response; PR, partial response; SD, stabledisease; PFS, Progression Free Survival; OS, Overall Survival; G, Gefitinib; E, Erlotinib.

Finally, we pooled data from 74 patients. Ages ranged from 30 to 82 years (median 58.5). Gender and smoking history were: male (33/74, 44.6%), female (41/74, 55.4%); never-smoker (50/72, 69.4%), smoker (22/72, 30.6%); There were 40 patients (40/74, 54.0%) with exon 19 deletion, 29 patients (29/74, 39.2%) with L858R mutation and 4 patients (4/74, 5.4%) with rare sensitive mutation (G719X, L861Q). One patient (1.4%) who had both a resistant mutation (T790M) and sensitive mutation (L858R) was excluded in the analysis of PFS and OS. Twenty-five (25/67, 37.3%) patients received erlotinib, 30 patients (30/67, 44.8%) received gefitinib, 11 patients (11/67, 16.4%) received icotinib and 1 patients (1/67, 1.5%) received dacomitinib. In 37 cases (37/66, 56.1%), EGFR-TKI was used as the first line of treatment while in 29 cases (29/66, 43.9%), second or more lines of treatment with EGFR-TKI were used. (**Table 1**).

There are 27 patients whose tumor responses were identified from published research. In total, 71 patients were evaluated for response. There were three patients with CR, 42 patients with PR, 16 patients with SD, and 10 patients with PD. It had an ORR of 63.4% (45/71) and DCR of 85.9% (61/71) (**Table 2**).

19 patients with PFS were identified in published research. In total, 63 patients were analyzed for PFS. All patients had a mPFS of 10.00 months (95% CI 6.73-13.27). Exon 19 deletion patients had a mPFS of 11.00 months (95% CI 6.70-15.30), while exon 21 L858R mutation patients had a mPFS of 10.00 months (95% CI 5.89-14.11) (P=0.771). Compared to rare sensitive mutations (G719X, L861Q) patients, exon 19 deletion patients or exon 21 L858R mutation patients had a longer mPFS (11.00 months vs. 2.10 months, *P*=0.005; 10.00 months vs. 2.10 months, *P*=0.019). Univariate analysis did not show significant correlations between the data sources, age, gender, smoking status, EGFR-TKI lines, and EGFR-TKI treatment and PFS. Multivariate analysis revealed no significant correlation between clinical features and PFS (**Table 5**).

**Table 5:** Association between clinical factors and the PFS.

	PFS (months)	Univariate analysis, *P* ^a^	Multivariate analysis, *P* ^b^
Data sources		0.257	0.279
Bicenter data	8.80		
Published data	11.00		
Age(years)		0.875	0.875
<60	9.00		
≥60	11.99		
Gender		0.467	0.442
Male	9.40		
Female	10.00		
Smoking status		0.907	0.863
Smoker (current/former)	9.40		
Non-smoker (never)	11.00		
EGFR mutation type		*P*1 = 0.005, *P*2 = 0.019, *P*3 = 0.771	0.181
19-DEL	11.00		
L858R	10.00		
G719X, L861Q	2.10		
Lines of EGFR-TKIs			0.127
1	8.05	0.116	
≥2	11.99		
EGFR-TKIs treatment		*P*4 = 0.101, *P*5 = 0.724, *P*6 = 0.087	0.864
Erlotinib	8.23		
Gefitinib	14.80		
Icotinib	11.77		

EGFR, epidermal growth factor receptor; TKI, tyrosine kinase inhibitor; PFS, progression free survival; ^a^, Log-rank test; ^b^, Cox regression test; P1, P (19-DEL vs. G719X, L861Q); P2, P (L858R vs. G719X,L861Q); P3, P (19-DEL vs. L858R); P4, P (Erlotinib vs. Gefitinib); P5, P (Erlotinib vs. Icotinib); P6, P (Gefitinib vs. Icotinib).

The data of OS was extracted in 18 patients from the published researches. The pooled analysis of OS included 62 patients. The mOS was 21.37 months (95% CI 16.01**-**26.73). Exon 19 deletion patients had a mOS of 23.67 months, while exon 21 L858R mutation patients had a mOS of 20.33 months (P=0.973). In univariate analysis, erlotinib treatment led to a longer OS compared with icotinib treatment (25.00 months vs. 15.01 months, P=0.061); In univariate analysis, a mOS of 23.67 months was seen in patients treated with gefitinib compared with 15.01 months in patients treated with icotinib (P=0.009); Univariate analyses showed no significant correlation between the data sources, age, gender, smoking status, and lines of EGFR-TKIs and OS. In multivariate analysis, no clinical features were found to be correlated significantly with OS (**Table 6**).

**Table 6:** Association between clinical factors and the OS.

	OS (months)	Univariate analysis, *P* ^a^	Multivariate analysis, *P* ^b^
Data sources		0.154	0.133
Bicenter data	19.43		
Published data	25.00		
Age (years)		0.125	0.100
<60	19.43		
≥60	28.97		
Gender		0.300	0.214
Male	27.50		
Female	21.37		
Smoking status		0.522	0.371
Smoker (current/former)	19.37		
Non-smoker (never)	21.37		
EGFR mutation type		*P*7 = 0.973, *P*8 = 0.064, *P*9 = 0.213	0.530
19-DEL	23.67		
L858R	20.33		
G719X, L861Q	–		
Lines of EGFR-TKI		0.422	0.500
1	20.33		
≥2	25.00		
EGFR-TKI treatment		*P*10 = 0.613, *P*11 = 0.061, *P*12 = 0.009	0.168
Erlotinib	25.00		
Gefitinib	23.67		
Icotinib	15.01		

EGFR, epidermal growth factor receptor; TKI, tyrosine kinase inhibitor; OS, overall survival; ^a,^ Log-rank test; ^b^, Cox regression test; P7, P (19-DEL vs. L858R); P8, P (19-DEL vs. G719X, L861Q); P9, P (L858R vs. G719X, L861Q); P10, P (Erlotinib vs. Gefitinib); P11, P (Erlotinib vs. Icotinib); P12, P (Gefitinib vs. Icotinib).

## Discussion

Literature studies concerning EGFR-TKI sensitivity in ASC harboring EGFR mutation are limited due to the low incidence of ASC in lung cancer. As a result, there is not enough evidence to support the efficacy of EGFR-TKI in the treatment of ASC. In our pooled analysis, there was an ORR of 63.4% and DCR of 85.9% in ASC patients treated with EGFR-TKI, and a mPFS of 10.00 months and a mOS of 21.37 months in these patients. Hence, ASC containing mutant EGFR are effectively treated with EGFR-TKI. Meanwhile, EGFR mutation can be detected in 54.8% of ASC patients which demonstrated that the mutation rate is parallel to ADC (8, 21). As EGFR mutations are highly prevalent in ASC patients and EGFR-TKIs are highly effective, we recommend routine EGFR mutation testing for all ASC patients. To our knowledge, our study represents one of the largest studies of EGFR-TKI efficacy in lung ASC patients harboring mutations in EGFR. We believe this data deserves clinical reference.

ADC has been successfully treated with EGFR-TKI in previous clinical studies, with ORRs of 70-85% and mPFS of 8-13 months (7, 22). Previous research has indicated that ASC patients with EGFR mutations achieve mPFS of 9.3 months when treated with first-generation EGFR-TKI (9). As a result of our study, lung ASC had an ORR of 63.4% and a median PFS of 10.00 months. Our study shows that lung ASC with EGFR mutations respond effectively to EGFR-TKI treatment, albeit with a slightly lower efficacy compared to pure adenocarcinomas. The distinct biological behavior of ASC, which includes both squamous and glandular components, might contribute to the differences in treatment outcomes (3). The heterogeneity within the tumor may impact the response to EGFR-TKI, as adenocarcinoma and squamous components may respond differently to treatment. Besides, the variability in the molecular profile of ASC tumors, as compared to pure adenocarcinomas, might also be a factor (8). This variability could influence the tumor’s response to EGFR-TKI therapy. Our study suggests a need for further research to explore the molecular mechanisms behind the differential response of ASC and pure adenocarcinomas to EGFR-TKI therapy.

According to our research, in patients with rare sensitive mutations (G719X, L861Q), the difference in PFS was statistically significant when compared to patients with deletion of exon 19 or exon 21 L858R mutations. However, because there were only 4 patients with rare sensitive mutation, the outcome needed to be further testified by more researches. Further, previous studies have shown that ADC patients with a L858R mutation in exon 21 of EGFR have significantly lower efficacy with EGFR-TKI treatment than patients in exon 19 of EGFR (23). However, patients with deletions in exon 19 and exon 21 L858R mutations had similar PFS (11.0 vs 10.0 months, P=0.771) and OS (23.67 vs. 20.33 months, P=0.973). The cause of this difference needs to further study. Our study primarily focused on the initial efficacy of EGFR-TKI in ASC patients. EGFR-TKI acquired resistance in lung ASC is gradually becoming a research hotspot (24). The progression and resistance mechanisms, including the frequency of T790M mutations, are undoubtedly crucial and future studies focusing on this aspect would indeed be valuable. Bsides, the efficacy in lung ASC of third generation TKI such as osimertinib and ceritinib still needs further study (25, 26). Immunotherapy has shown promising prospects in the treatment of lung ASC (5).

In one published research, 55 ASC patients were demonstrated dual differentiation with varying proportions of ADC and SCC by using the microdissection (27). There is a pity that pathology was unable to determine which of 44 patients carried squamous cell carcinomatous and adenocarcinomatous components. Moreover, some researches discovered that the identical EGFR mutation patterns in the squamous cell carcinomatous and the adenocarcinomatous components in each patient, indicated the monoclonality of the two tumor components in ASC patients (28, 29). This conclusion was also testified by other researches (3). Since the identical EGFR mutation patterns occurred in the squamous cell carcinomatous and the adenocarcinomatous components of ASC, It may be the proportion of two tumor components in EGFR mutant ASC patients that determines the efficacy of EGFR-TKI in EGFR mutant ASC patients. The predominance of one component over the other could potentially affect the treatment outcomes. In addition, the therapeutic advantages on adenocarcinoma components of TKI may generate the withering of the adenocarcinomatous components of ASC, while the squamous cell carcinomatous of ASC gain a quantitative advantage (30). Researchers at our cancer center are investigating how the ratio of these two tumor components and EGFR-TKI efficacy are related.

In NSCLC, especially in metastatic disease, small biopsy samples can make it difficult to accurately differentiate between squamous cell carcinoma and ASC (1). This distinction is crucial as it impacts treatment decisions. Molecular testing, including EGFR mutation analysis, can play a critical role in identifying patients who might benefit from targeted therapies (31). This is particularly relevant in cases where histological classification is uncertain. Given the histological overlap between squamous tumors and ASC, molecular testing provides a more precise approach to identify the tumor’s characteristics, thus guiding appropriate treatment (3). The American Society of Clinical Oncology (ASCO) emphasizes the need for comprehensive molecular profiling in NSCLC. By incorporating molecular testing, clinicians can better tailor treatment strategies to individual patient needs, especially for those with rare or atypical NSCLC subtypes like ASC.

It is necessary to illustrate the limitations of this study. Among the selected published studies, inclusion criteria and test methods for EGFR mutations were different, and clinical traits were not completely described. Moreover, the retrospective nature was another limitation of this research. The low incidence of ASC in lung cancer, however, makes our research quite significant as well.

In conclusion, this study which involved all available data, including data collected from our cancer centers of China and that pooled from previous studies, and identified the clinical profiles of EGFR-TKI application, suggested that EGFR-TKI was found to be an effective treatment in ASC harboring mutations in EGFR. Furthermore, the study recommends that EGFR mutation testing be conducted routinely on all lung ASC patients.

## Data availability statement

The original contributions presented in the study are included in the article/supplementary material. Further inquiries can be directed to the corresponding author.

## Ethics statement

The studies involving humans were approved by West China hospital’s Institutional Review Board. The studies were conducted in accordance with the local legislation and institutional requirements. Written informed consent for participation in this study was provided by the participants’ legal guardians/next of kin.

## Author contributions

XX: Conceptualization, Data curation, Writing – original draft. WD: Data curation, Writing – original draft. YZ: Supervision, Validation, Writing – original draft. YYL: Investigation, Software, Writing – original draft. MY: Formal analysis, Methodology, Resources, Writing – original draft. YML: Writing – review & editing.
